# LitSumm: large language models for literature summarization of noncoding RNAs

**DOI:** 10.1093/database/baaf006

**Published:** 2025-02-05

**Authors:** Andrew Green, Carlos Eduardo Ribas, Nancy Ontiveros-Palacios, Sam Griffiths-Jones, Anton I Petrov, Alex Bateman, Blake Sweeney

**Affiliations:** European Molecular Biology Laboratory, European Bioinformatics Institute (EMBL-EBI), Wellcome Genome Campus, Hinxton CB10 1SD, UK; European Molecular Biology Laboratory, European Bioinformatics Institute (EMBL-EBI), Wellcome Genome Campus, Hinxton CB10 1SD, UK; European Molecular Biology Laboratory, European Bioinformatics Institute (EMBL-EBI), Wellcome Genome Campus, Hinxton CB10 1SD, UK; School of Biological Sciences, Faculty of Medicine, Biology and Health, Michael Smith Building, The University of Manchester, Manchester M13 9NT, UK; Riboscope Ltd, 23 King St, Cambridge CB1 1AH, UK; European Molecular Biology Laboratory, European Bioinformatics Institute (EMBL-EBI), Wellcome Genome Campus, Hinxton CB10 1SD, UK; European Molecular Biology Laboratory, European Bioinformatics Institute (EMBL-EBI), Wellcome Genome Campus, Hinxton CB10 1SD, UK

## Abstract

Curation of literature in life sciences is a growing challenge. The continued increase in the rate of publication, coupled with the relatively fixed number of curators worldwide, presents a major challenge to developers of biomedical knowledgebases. Very few knowledgebases have resources to scale to the whole relevant literature and all have to prioritize their efforts.

In this work, we take a first step to alleviating the lack of curator time in RNA science by generating summaries of literature for noncoding RNAs using large language models (LLMs). We demonstrate that high-quality, factually accurate summaries with accurate references can be automatically generated from the literature using a commercial LLM and a chain of prompts and checks. Manual assessment was carried out for a subset of summaries, with the majority being rated extremely high quality.

We apply our tool to a selection of >4600 ncRNAs and make the generated summaries available via the RNAcentral resource. We conclude that automated literature summarization is feasible with the current generation of LLMs, provided that careful prompting and automated checking are applied.

**Database URL**: https://rnacentral.org/

## Introduction

Curation in life sciences is the process by which facts about a biological entity or process are extracted from the scientific literature, collated, and organized into a structured form for storage in a database. This knowledge can then be more easily understood, compared, and computed. The curation task is a time-consuming and often challenging one in which subject matter experts triage literature, select curatable papers, and review them for the rich information they provide about a given biological entity [1]. Researchers search curated databases (knowledgebases) for information about the entities they are studying and incorporate curated facts into the design of their next study, which may in turn be curated. This virtuous circle is fundamental to the functioning of research in life sciences.

One of the most basic requirements for a researcher is a broad understanding of the molecule of interest. A broad overview is most easily gained from a short summary of the literature. Such summaries are often produced as part of the curation process, for example, UniProt [2] gives an overview of a protein’s function on its protein entry pages. Similarly, some model organism databases have curator-written descriptions of the genes they contain (e.g. Saccharomyces Genome Database [3] and FlyBase [4]). Summaries are time-consuming to produce because there may be a large amount of disparate information to synthesize; because of the difficulty, many databases still do not yet have summaries for all the entities they contain, e.g. the RNAcentral database does not contain summaries for ncRNAs. In addition, human-written summaries are prone to become outdated due to the lack of available curator time.

There are a limited number of curators in the world and the rate of publication and the complexity of the research papers continues to increase. The mismatch between the effort that is required and that which can be applied has led many to use computational techniques at all stages of curation. Natural processing (NLP) has been applied for many years, with cutting-edge techniques being used as they become available. However, to date these approaches have had limited success. Recently, language models, and in particular LLMs, have attained sufficient quality to be applicable to curation. Recent efforts have used LLMs to summarize gene sets [5], mine knowledge from synthetic biology literature [6], and other tasks previously done by NLP methods [7]. In most cases, LLMs are able to perform remarkably well with little or no fine-tuning training data, opening the potential for their application in resource-limited fields.

One field in which the lack of curation effort is particularly acute is ncRNA science. ncRNAs are any RNA transcribed in the cell that does not encode a protein. ncRNAs are critical to the functioning of the cell by forming the core of the ribosome, splicing pre-mRNAs in the spliceosome, and regulating gene expression through microRNAs (miRNAs), long ncRNAs (lncRNAs), small nucleolar RNAs (snoRNAs) and many other RNA types. However, as a field, ncRNA has very little curation resource compared to the field of proteins. Rfam [8] and RNAcentral [9] are two of the primary databases in RNA science. Rfam is a database containing over 4100 RNA families, while RNAcentral is the ncRNA equivalent of UniProt containing over 30 million sequences at the time of writing. Rfam includes curated descriptions of each RNA family. These descriptions are quite general as they describe the function across all organisms in which the family is found. RNAcentral imports data from other resources and as of release 22, it contains data from 52 other resources, of which 12 provide curated data. RNAcentral has previously made efforts to connect users with the relevant literature with the development of the LitScan tool (described in detail in the Sentence acquisition section below) to explore the EuropePMC API and extract citations and relevant sentences from the literature. However, LitScan still lacks a way to provide a coherent and comprehensive overview of an RNA. As a comprehensive source of information on ncRNA, the RNAcentral database is a natural location for the development of tools to more easily connect users with the ncRNA literature.

In this work, we apply a tool based on GPT4, developed by OpenAI, to produce automated summaries for a large number of ncRNA genes. Summaries are generated from sentences mentioning ncRNAs extracted from the literature and displayed on the RNAcentral website. We detail our approach to sentence acquisition by exploring the EuropePMC API to allow the extraction of relevant passages. These snippets are then passed through a pipeline of selection, summarization, automated checking, and automated refinement when necessary, which we named LitSumm. The output of this is 4618 summaries detailing the literature relating to ∼28 700 transcripts. A randomly selected subset of 50 summaries representative of RNA type and context size are manually evaluated by four expert raters.

## Materials and methods

### RNA selection

To keep costs and computation size within reasonable limits, we focus on a subset of RNAs of broad interest to the community. We include RNAs contributed by the HUGO Gene Nomenclature Committee (HGNC) [10], miRBase [11], mirGeneDB [12], and snoDB [13] databases. Within these, we identify primary identifiers and aliases as supplied by the source database.

A large fraction of the RNAs we consider are miRNAs that are associated with a large corpus of scientific literature. Many of these are referred to by identifiers that are not organism specific such as ‘mir-21’. Having nonspecific identifiers leads to a very large number of papers that must be summarized across a diverse range of organisms; this can lead to confusing or inaccurate statements about the function of an miRNA in a given organism when the function was actually observed elsewhere. More recently, identifiers including an indication of the species have become more common, in this case for example ‘hsa-mir-21’ for the human-specific miRNA. The difference in the number of papers discussing these identifiers is enormous. To ensure the specificity of summaries, we restrict the IDs used to generate summaries of miRNAs to only those specific to a species. The exception to this rule is for human miRNAs coming from HGNC, which often have identifiers like ‘MIR944’ and are included in the set of ncRNAs we summarize.

### Large language models

LLMs are a class of machine learning models that have very large numbers of parameters, hundreds of billions is common, and are adept at predicting the most probable next token given an input sequence. LLMs are built on the transformer architecture [14]. This architecture imposes several limits: first, it is expensive in terms of memory and computation to operate on large amounts of text. This imposes a limit on the amount of text, called a context length. Secondly, LLMs do not operate on words, but instead on tokens. This means that context lengths are always given as the number of tokens that can be fed into a model; a helpful rule of thumb is that a token is ∼0.75 words, so a 4096 token context would be ∼3000 words [15].

In this work, GPT4-turbo (https://platform.openai.com/docs/models/gpt-4-and-gpt-4-turbo), an LLM provided by OpenAI, is used. Specifically, we use the gpt-4-1106-preview model through the OpenAI API. The primary parameter controlling the text generation is temperature, *T*, which alters the sampling distribution of the next token; *T *= 0 would make the model only choose the most likely next token, while higher values allow the model to explore the distribution of the next token. We use a relatively low *T *= 0.1 (default *T *= 1), a balance between determinism and flexibility to rewrite parts of the context into a coherent summary. Low *T* also reduces the likelihood of model ‘hallucinations’, a common problem where the LLM will invent facts [16].

Two other parameters used to control the generation of the model are the presence and frequency penalties. These alter the sampling distribution by adding a penalty to tokens already present in the text to reduce repetition. They can also be used to encourage reuse by giving negative values. We use a presence penalty of −2 in the initial summary generation call to ensure the model restates tokens from the context in the summary, but with a frequency penalty of 1 to avoid repetition. All operations involving the LLM are abstracted using the LangChain python library (https://github.com/hwchase17/langchain).

### Sentence acquisition

To gather what is being said about an RNA in the literature, we explore the EuropePMC API using a query designed to find articles discussing ncRNA while minimizing false positives. The query used is ‘query=(“<RNA ID>” AND (“rna” OR “mrna” OR “ncrna” OR “lncrna” OR “rrna” OR “sncrna”) AND IN_EPMC:Y AND OPEN_ACCESS:Y AND NOT SRC:PPR)’, with the collection of terms in parentheses aiming to filter out false positives that mention the ID but not a type of ncRNA; the query also explicitly requires open access and excludes preprints. We restrict this search to the open-access subset at EuropePMC such that we can access and reuse the full text, aside from this no other restrictions are placed on the articles we retrieve or use. RNAcentral’s comprehensive and regularly updated collection of cross-references between RNA resources enables us to identify papers that refer to the same RNA using different names or identifiers.

Once articles about an RNA have been identified, the full text is retrieved and searched to (I) validate that the ID is mentioned in the article and (II) extract sentences that mention the ID. The identified article PMCIDs and contained sentences are stored in a database at RNAcentral. The results of this can be seen on RNAcentral, where the tool is referred to as LitScan (https://rnacentral.org/help/litscan) and has an interface allowing users to explore the results (e.g. https://rnacentral.org/rna/URS000075D66B/9606?tab=pub). In this work, we use LitScan as a source of statements about RNAs, which can be used to provide an overview of the literature about them.

### Sentence selection

Not all ncRNAs are studied equally. For many, we know about their existence only because they have been sequenced and deposited in sequence archives such as the European Nucleotide Archive [17]; for these RNAs, we have no papers to summarize. A significant subset of RNAs appear in only a few articles where their existence is established, and occasionally, some aspect of function, localization, or other information is determined. To ensure a reasonable amount of information for the LLM to summarize, we restrict the lower bound of sentence counts to five. These five sentences could come from a single paper, which allows summarization of single papers that present the only source of information about an RNA.

Above this threshold, there are two factors driving the selection of sentences from which to summarize: context length and information coverage. For LitSumm, we restrict ourselves to a 4096 token context and impose a limit of 2560 tokens (∼1920 words) in the context to allow for prompting and revisions. We limited ourselves to a 4096 token context for two reasons: feasibility of downstream finetuning of an open LLM and cost—using the full 128k token window of GPT4 would be prohibitively expensive, since API calls are charged per token. The limit of 2560 is arrived at by considering the length of output summaries (∼255 tokens), prompts (60–140 tokens), and the number of tokens required to send a summary for revision (∼1150) for a random subset of RNAs. For the majority of ncRNAs, the total available sentences fall within this context limit, so no selection is applied beyond the five-sentence lower limit.

For some ncRNAs such as well-studied miRNAs (e.g. hsa-mir-191) and snoRNAs (e.g. SNORD35A) among others, totalling 1704 RNAs, we find too many sentences to use them all, meaning that a selection step is necessary. To select sentences, we apply a topic modelling approach [18]. We used the SentenceTransformers package [19], with the pretrained ‘all-MiniLM-L6-v2’ model, which embeds each sentence into a 384-dimensional vector, the dimensionality of its output layer. Then, the Uniform Manifold Approximation and Projection dimensionality reduction technique [20] is applied to reduce the vector dimension to 20, and the HDBSCAN clustering algorithm [21] produces clusters of similar sentences. While 384 is not a particularly high dimensionality, it has been shown that reducing dimensionality significantly improves clustering performance across a variety of tasks [22]; we choose a dimensionality of 20 such that we can use the fast_hdbscan python library (https://github.com/TutteInstitute/fast_hdbscan), for which 20D is the maximum recommended dimensionality. Cluster exemplars were sampled in a round-robin fashion until the context was filled to ensure a broad coverage of topics. In the case where all exemplars did not fill the context, sentences were sampled from the clusters themselves in the same round-robin way.

An important minority of ncRNAs are very heavily studied. These include ncRNAs like XIST, MALAT1, and NEAT1, each of which appears in thousands of articles. In these cases, our selection technique still results in too many tokens, so we apply a greedy selection algorithm to the cluster exemplars. An exemplar is selected in the largest cluster, then the vector embedding is used to calculate the similarity to all exemplars in other clusters. The exemplar least similar to the selected exemplar is selected, and the process continues by evaluating the distance from all selected exemplars. The process repeats until the context is filled.

For some RNAs, there were too many sentences to use all, but not enough to apply the topic modelling approach. In this case, the sentences were sorted in descending order of tokenized length, and the first *k*-sentences were taken such that the context was filled.

In all cases, no criteria are applied to the selection of sentences to use in building the summary beyond them having a direct mention of the RNA being summarized.

### Prompts

One of the most critical criteria for a scientific summary is that it contains only factual information. Additionally, tracing the provenance of statements in the summary is important for verifiability. We have designed a chain of prompts through iterative refinement on a subset of examples with these objectives in mind. The first prompt generates the summary, and if there are problems, subsequent prompts attempt to guide the LLM into rectifying them.

**Figure 1.**
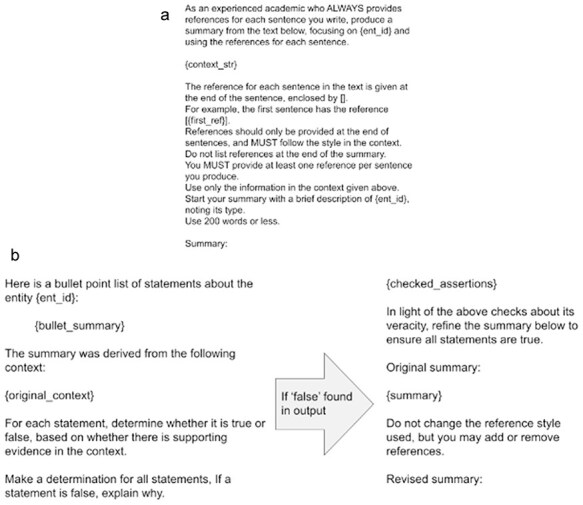
(a) The initial prompt used to generate a first-pass summary from the generated context. Variables are enclosed in {} and are replaced with their values before sending the prompt to the LLM. (b) Prompts used for the self-consistency checking stage including inaccurate statement detection and revision. All prompts are reproduced as plain text in Appendix A in the Supplementary material.

The first prompt is shown in Fig. 1a.

Here, the model is instructed several times to use references, and the style of reference desired, with an example. The LLM is further instructed not to use ‘external sources’; this aims to stop the LLM inserting any facts that are not present in the context. While these facts could be accurate, there is no way of finding out where they come from, and they risk being inaccurate, which we try to avoid at all costs. These instructions, combined with the sampling parameters, reduce the likelihood of the model inserting facts not present in the context.

After the summary is returned from the model, references are evaluated. This consists of five checks, any one of which can trigger a regeneration of the summary. The five checks are as follows:

1. Adequacy of references: are there enough references for the number of sentences in the summary? We require at least 0.5 references per sentence.
2. Formatting of references: We require the model to cite sentences by using PubMed Central identifiers (formatted like PMCXXXXXX).
3. Realness of references: Are all the references in the summary present in the context? This should catch cases where the model has invented a PMCID.
4. Location of references: references should be at the end of sentences usually. This is intended to stop the model from putting all references at the end of the summary and not indicating which statement comes from which reference.
5. Number of references per instance: this check catches the model putting many PMCIDs into a single pair of brackets, which is undesirable for the purposes of provenance checking. We require no >50% of the total number of references in any given citation.

Each check has a specific ‘rescue’ prompt that is applied when the summary makes a particular mistake. There are four of these, shown in Fig. A1 in the Supplementary material. To keep costs and computation time within four hundred dollars and a total run-time of 1 day, a maximum of four attempts are given to produce a summary. If the summary is still not produced after these attempts, it is flagged as potentially problematic. However, if the summary passes the check within the70 limit of four attempts, it continues to the next stage.

Once all reference-based checks have passed, the accuracy of the summary is evaluated. To do this, the summary is broken into a bulleted list and provided alongside the original context. The model is instructed to state whether each bullet is true or false based on the context and to find the support in the context. Importantly, we not only ask for a true/false but also ask for an explanation of why. When a summary contains a misleading or false statement, the output of this step, along with the summary, is fed back to the model which is instructed to amend the summary accordingly. These two steps combine approaches to LLM self-fact-checking [23] and chain-of-thought prompting [24]. In combination, these improve summaries. The prompts used in these stages are shown in Fig. 1b.

**Figure 2.**
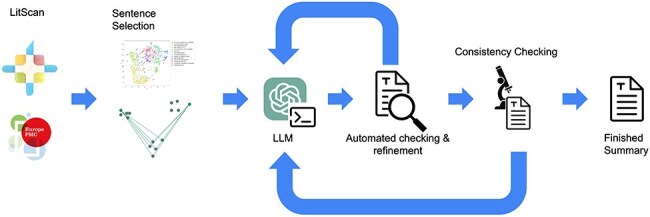
A flow diagram of the whole LitSumm tool. Information from the EuropePMC API flows from the left to the right, through a sentence selection step before several rounds of self-checking and refinement. Finished summaries are written to disk before being uploaded to the RNAcentral database enmasse.

Once this stage is complete, the summary is given a final reference check, and if successful the summary is considered finished. An overview figure of the whole LitSumm tool is shown in Fig. 2.

### Human and automated assessment

To evaluate the quality of the output, a subset of the summaries was assessed in parallel by four reviewers. These reviewers were chosen from the coauthors and were specifically selected to represent diverse academic backgrounds, including expertise in data curation, RNA biology, and machine learning. A subset of 50 summaries was randomly selected, stratified by context length, and loaded into a web platform to provide feedback. Figure B1 in the Supplementary material shows a screenshot of the web platform used. The distribution of summaries over RNA types is shown in Table 1.

**Table 1.** Distribution of RNA types for the 50 summaries reviewed

RNA type	Count
lncRNA	23
pre_miRNA	11
miRNA	10
snoRNA	4
Other	2

Since we primarily sampled according to the total number of tokens available for each RNA, without considering the type, this distribution broadly follows the distribution of RNA types in the whole dataset.

Summaries were presented alongside the context from which they were generated to allow the raters to evaluate the claims made in the summary. The ratings were given on a 1–5 scale based on the rubric shown in Table B1 in the Supplementary material. Briefly, a rating of 1 would indicate multiple serious problems with a summary (fake references, inaccurate statements, etc.); a rating of 2 indicates at most two misleading/incorrect statements or one serious error; a rating of 3 indicates an acceptable summary with at most one minor misleading/incorrect statement; a rating of 4 indicates a summary with no incorrect/misleading statements but with other problems such as poor flow; and a rating of 5 would indicate an excellent summary (all statements referenced and true, good flow, etc.). All summaries rated 3 and above must have correct, adequate references. Raters were asked to score a summary based only on the information in the context, not using any extra information from the linked articles, or their own knowledge. We also provided a series of tickboxes designed to identify particular failure modes, these are also shown in Table B2 of the Supplementary material.

Raters 0–2 were involved in the design of the rubric and had the same training with the tool. Experience in RNA science differed between the raters, with Raters 0 and 1 being more experienced than Rater 2, and Rater 0 being a professional curator. Rater 3 was not involved in the development of the summarization or assessment tools and received written training to use the rubric before completing their rating session.

## Results

We focused on RNAs from authoritative databases including HGNC, miRBase, mirGeneDB, and snoDB, with particular attention to unambiguous identifiers to ensure accurate functional attribution. From these databases, an initial set of 4618 RNA identifiers were selected for summarization. Our pipeline extracted relevant sentences from open-access papers in EuropePMC using RNA-specific search queries. Representative sentences were selected using topic modelling and sampling to maintain information diversity. These selected sentences were then processed through GPT-4-turbo using a prompt chain that enforces factual accuracy and proper citation. Each generated summary underwent multiple rounds of self-consistency checking and refinement, validating both reference formatting and factual accuracy against the source material. A subset of the final summaries was evaluated by a panel of experts across multiple domains, including RNA biology, data curation, and machine learning.

**Figure 3.**
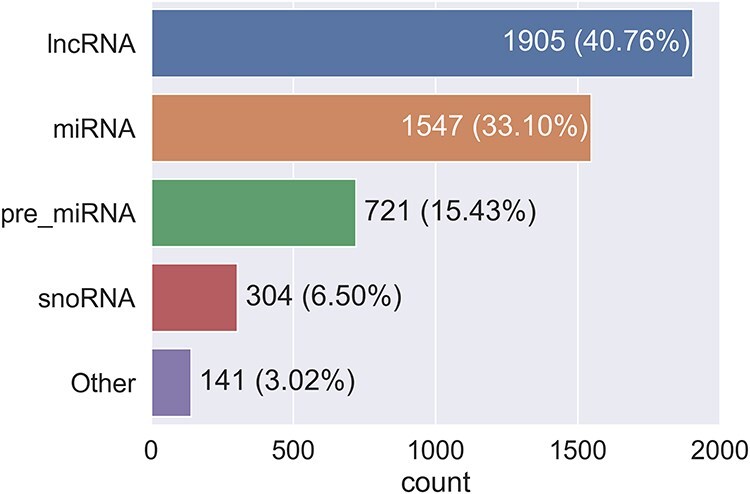
The distribution of RNA types selected for summarization.

The 4618 RNA identifiers selected for summarization represent a coverage of ∼28 700 transcripts and 4605 unique RNA sequences in RNAcentral, and ∼177 500 papers containing the identifiers. The distribution of RNA types is shown in Fig. 3.

The majority of RNAs come from the RNA-type-specific databases miRBase, mirGeneDB, and snoDB, which provide miRNAs and snoRNAs; all lncRNAs come from HGNC and are therefore only those found in humans. The small number of ‘other’-type RNAs is from HGNC, including, for example, rRNAs, RNAses, and some RNAs with imprecise-type labels such as the generic ncRNA. As expected from the chosen databases, the majority of the RNAs selected are human, with nonhuman RNAs coming primarily from miRBase and mirGeneDB.

**Figure 4.**
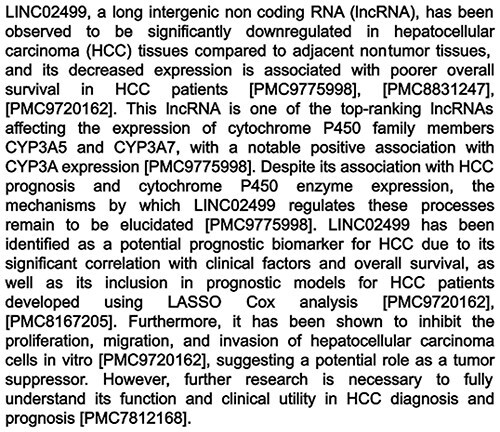
Example summary generated by the tool. This example is an lncRNA, examples for other RNA types can be found in Appendix C of the Supplementary material.

The full generation process for each summary, including the automated checking, consistency checking, and all revisions, took on average 29 s and cost $0.05. These values are estimated from the total time to generate all 4618 summaries, and the total cost across the generation period as recorded by OpenAI for billing. An example summary is shown in Fig. 4, and all summaries can be browsed by going to the RNAcentral website and searching ‘has_litsumm:“True”’ (https://rnacentral.org/search?q=has_litsumm:%22True%22). We also make the entire dataset of contexts and their summaries available online at https://huggingface.co/datasets/RNAcentral/litsumm-v1.5.

Automated checking, primarily of reference adequacy and accuracy, identified problems in 2.1% of summaries, which were adequately rectified within the four allowed revisions in 76% of cases, meaning that overall 99.5% of summaries passed our automated checks. The self-consistency check identified problems in 17.3% of summaries, which were rectified in 51% of cases giving an overall pass rate of 91.5%. The pass rates at each stage are shown in Table 2.

**Table 2.** Pass rates of the automated checking and self-consistency checking stages in the LitSumm pipeline

Failure mode	Pass rate (%)	Number of passing summaries
References—first pass	97.9	4519
References—after revision	99.5	4594
Self-consistency—no problems found	82.7	3820
Self-consistency—no problems after revision	91.5	4226

The table shows the percentage and number of summaries that pass each stage, including revision stages.

**Figure 5.**
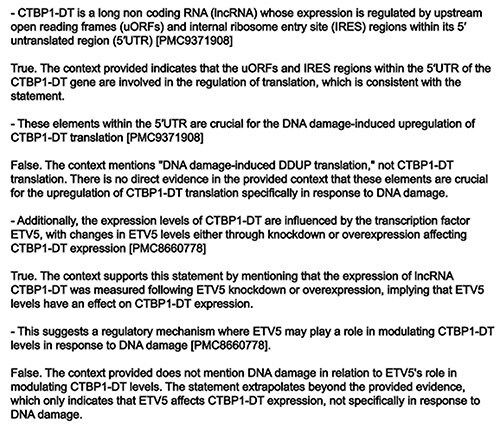
Example output of the veracity checker. In this case, CTBP1-DT presents two sentences validated as TRUE and two FALSE sentences. The offending sentences have been removed by the model in the final summary.

An example of the type of error identified and rectified by the consistency check is shown in Fig. 5.

**Figure 6.**
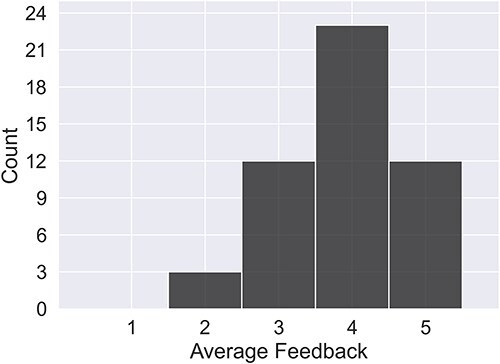
The average rating per summary across all raters. Note that Rater 3 gave scores only for a subset of 21 miRNAs.

Human evaluation was carried out for a subset of randomly sampled RNAs. These RNAs cover the full range of context sizes and ncRNA types. The subset consists of 50 RNAs in total, for which three raters assessed quality. Of these 50 RNAs, 21 (covering only miRNA) were also scored by an expert in miRNA (Rater 3). The human rating, on a scale of 1–5 with 5 being excellent and 1 indicating the presence of some serious failure, is shown for all raters in Fig. 6.

From the human ratings, 94% of summaries were rated good or excellent. In the majority of cases where a summary was rated inadequate (score of 2 or less), the problem identified by the raters had to do with poor synthesis of facts from multiple sources not caught by the automated consistency check, or reference misattribution, where a reference for a given sentence does not match the information content or is irrelevant. Reference errors are penalized strongly in the marking rubric, as they are misleading statements. LLMs are known to struggle to accurately combine facts across different documents [25]. This has been observed in previous studies of multi-document summarizations and may be connected to input construction. A summary of the failures identified is shown in Table 3, and the best- and worst-rated summaries per RNA type are shown in Appendix C in the Supplementary material.

**Table 3.** Reasons given for poor rating in those cases where a rating <3 was given

Rater	Total (out of 50 summaries)	Reference formatting	Hallucination/false info	Other
Rater 0	9	4	9	1
Rater 1	5	0	3	2
Rater 2	7	3	3	3
Rater 3	3/21	0	1/21	4/21

Summaries rated inadequate by human inspection are retained, but not displayed on RNAcentral. Similarly, where problems were identified by automated checks, e.g. failing to insert correct references, or automatically detected self-consistency errors, the summaries are retained but not displayed. This means that from a total of 4605 unique RNA sequences, 4602 have a displayed summary on the RNAcentral website.

Additionally, we observed instances of reference misattribution, where unrelated or incorrect references were added to sentences, undermining the ease of verification of the summaries. Furthermore, the model exhibited a tendency to over-infer and make unwarranted connections, often overstating the significance of findings, such as prematurely identifying biomarkers. We also noted cases of unsupported expansions, where the model introduced information that was not supported by the original text, for example, expanding DFU as ‘Diabetic Foot Ulcer’ when the provided context made no mention of diabetes, feet, or ulcers. Finally, issues with flow and coherence were evident, including the inclusion of internal instructions and inappropriate recommendations. These findings highlight the need for continued refinement and evaluation of LLMs in generating accurate and reliable summaries of scientific literature.

Summaries are available on RNAcentral by exploring results of the search ‘has_litsumm:“True”’ (https://rnacentral.org/search?q=has_litsumm:%22True%22) or by exploring the ‘Literature Integration’ facet and selecting ‘AI generated summaries’. We also make the entire dataset of contexts and their summaries available online at https://huggingface.co/datasets/RNAcentral/litsumm-v1.5. Summaries will be updated with each RNAcentral release by rerunning the LitSumm pipeline, including rerunning LitScan to identify new literature; future releases may move to the use of a local model. Previous versions of the summaries will be versioned and made available, including metadata on the generation, such as the LLM model and sentence selection techniques used. Future work will include moving to a fine-tuned local model, which will allow expansion to more ncRNAs and a more even taxonomic coverage without significant additional cost.

## Discussion

In this work, we present an application of LLMs to perform literature curation for ncRNA. We show that a pipeline with a series of automated checks and carefully designed prompts can produce high-quality literature summaries. We also demonstrate techniques to minimize untrue information and ensure high-quality referencing in the summary.

Human ratings of a representative subset of the summaries generated have been collected and show that the majority of summaries are of high or very high quality, with a small number of common failure modes. The identified failure modes primarily fall into two categories: relating to referencing and relating to information synthesis/inference from multiple sources.

The size of the fully annotated set is small, at only 50 summaries, but thorough, with three of the raters rating all 50 summaries. This is in line with the sizes of other multi-rated summarization datasets [26], but with a greater overlap (100% vs. 20% in Wang *et al*.). Collecting multiple ratings for each summary improves our sensitivity to nuanced errors at the cost of coverage. The failure modes we identified were consistent across instances and were picked up by all raters; however, more nuanced errors were identified in context-specific cases and were not picked up by all raters. We are confident that our ratings identified the nuanced errors made by the LLM, but given the small coverage, identifying a clear pattern to these errors is difficult. It may be preferable to allocate rating time differently to maximize coverage with some minimal overlap to assess consistency.

In this work, the human ratings have been performed by four of the coauthors of the paper for all summaries. Having authors’ rate summaries introduces a conflict of interest in that the authors have a vested interest in the ratings being ‘good’. To mitigate the potential for bias, we employed a grading rubric and multiple rating for all summaries and further asked an external expert to rate summaries in their own field of expertise (also a coauthor). We believe the bias is minimal, evidenced by the low ratings for poor-quality summaries given by all raters. In future, it would be preferable to have additional independent ratings.

Summarization is a task that has been approached by language models previously, such as the T5 architecture [27]. While these models do perform well on summarization and other tasks, they are not as general purpose as modern, instruction-tuned LLMs, which are often equally adept at summarization. As such, basing the LitSumm architecture on a single driving model simplifies the tool. LLM-driven summarization has been done in several other fields. For example, Joachimiak *et al*. developed a similar tool, SPINDOCTOR, which is used to generate a summary from gene descriptions; the summary is then used in a gene enrichment analysis [5]. Joachimiak *et al*. evaluate the results of their gene enrichment against standard tools and find that their method is comparable, although it misses some important terms. SPINDOCTOR differs from LitSumm in that the input is a set of genes known to be enriched in an experiment, and the output is a summary of their commonalities, whereas LitSumm produces a broad overview summary of a single RNA from many literature-derived statements. SPINDOCTOR also does not need to assess the consistency of their summary with the context from which it is generated, and does not give the provenance of statements, since their input is human-derived.

One field in which similar considerations have to be made is medicine, where the accuracy and provenance of statements are paramount. Shaib *et al*. evaluate GPT 3 for the summarization and synthesis of many randomized controlled trial reports. They find that while the LLM produces coherent summaries, it often fails to synthesize information from multiple sources adequately and may be over-confident in its conclusions [28]. In our evaluation, we find similar failure modes, where the model misunderstands statements where it tries to synthesize information from more than one source.

A key aspect of our pipeline is the use of self-consistency checking and revision using chain-of-thought prompting. These two concepts have been applied in other contexts, such as question answering over documents [29], but have yet to be applied to literature curation. Despite our best efforts to reduce hallucinations and ensure wholly factual summaries, ∼17% of cases still have some problems, indicating the need for consistency checking. Feeding the output of the self-checking back into the model reduces this to 8.5%, which is encouraging, but also indicates the need for human intervention in this complex field where LLMs still struggle to fully comprehend scientific literature. In particular, the consistency check developed here is not effective at identifying inferences made by the LLM that are incorrect, because there is ‘indirect’ support in the context. There is also the possibility of ‘feedback hallucination’ in which the generated instructions to rescue a summary contain a hallucination, which if not checked will be inserted by the LLM as it follows its own instructions. As LLMs become stronger and have more advanced reasoning capabilities, this will become an increasingly problematic failure mode; the detection of errors of this sort is an area of active research in the NLP field generally.

We investigated the observed errors in the produced summaries, finding that the majority of poorly rated summaries share several common problems. Namely, we observe incorrect reference attribution, the inclusion of irrelevant details while missing important information, and statements unsupported by the context as the most common problems. More details of this error analysis can be found in Table B2 and Appendix C in the Supplementary material.

Another limitation relates to the literature itself and here is primarily seen with miRNAs. Many gene names or identifiers are ambiguous in that they can be used to refer to multiple organisms or may conflate the mature product and precursor hairpin. We have restricted ourselves to species-specific IDs (e.g. hsa-mir-126), meaning that the generated summaries should be consistent and limited to a single organism, but a significant fraction of the literature does not use these IDs. Thus, we are missing information. We could use a broader set of identifiers, but then we must be able to distinguish which species is being discussed in each paper. There are ways this could be addressed—for example, using the ORGANISMS database [30] to identify which organism a given article is about and then using this information to produce organism-specific summaries, despite the usage of nonspecific terms. However, the accuracy of such resources is questionable, meaning that we do not know which organism a paper discusses at present. We leave this problem as future work.

Other automated assessment methods have been developed that use another LLM to give a rating to the output of an LLM. This can be done with very little guidance as in single answer grading, described by Zheng *et al*. [31], where GPT4 is simply asked to grade output, or in a much more guided and structured way as in Prometheus [32], where a gold standard answer and marking rubric are provided to the model. While LLM rating approaches have not been applied here, such a tool would be valuable to ensure the quality of summaries without extensive human curation. However, the development of a suitable rubric is not straightforward; we plan to approach this problem in future work.

One limitation that will be difficult to address is the openness of literature. Our sentences come from the open-access subset of EuropePMC. While this data source is growing as more authors publish open access, it still does not allow access to the majority of knowledge, particularly that from earlier decades. Many knowledgebases make extensive use of closed-access literature in their curation; their primary concern is the quality of the information being curated, not the availability of the information; therefore, the open-access status of a paper is not an impediment to its being curated. However, the inability of this tool, and those which will doubtless come after it, to use closed-access literature does highlight the need for authors, institutions, and funders to push for open-access publication with a permissive licence for reuse.

Often there is too much literature available to feed all of it into an LLM to generate a summary. Recently, LLMs have been getting considerably larger context sizes, for example, GPT4 can now accept up to 128k tokens. However, this is unlikely to be a solution in itself; LLMs do not attend to their entire context equally [33], and having a larger context and expecting the LLM to use it all are unlikely to work, although some recent work has shown that this may be soluble [34]. In this work, topic modelling is used to reduce the amount of text to be summarized. This introduces problems related to the context construction that lead to inaccurate sentences being generated by the LLM. Worse, the automated fact-checking is blind to this type of failure, due to there being ‘evidence’ in the context which supports the inaccurate sentence. Therefore, we would recommend against the use of topic modelling alone to generate input context for an LLM, since it likely introduces more problems than alternative approaches such as vector store-based retrieval augmented generation (RAG). A better approach may be to decompose the summary into sections and apply a RAG [35] approach to each in turn by applying semantic search for only passages about, for example, expression.

The field of LLM research is moving extremely rapidly and we expect that significant improvements will be possible in our pipeline simply by adopting newer LLM technology. Our current work is based on GPT4, having originally been developed with GPT3.5; this allows us to see the improvement in LLM technology, which we show in Appendix D in Supplementary material with a brief A/B preference test between GPT3.5 and GPT4, and examination of pass rates through our pipeline. Moving to openly available models could enable future work on fine-tuning the LLM for the biological summarization task.

## Conclusion

In conclusion, we have demonstrated that LLMs are a powerful tool for the summarization of scientific literature and, with appropriate prompting and self-checking, can produce summaries of high quality with adequate references. Using the tool developed here, 4618 high-quality summaries have been provided for RNAcentral, providing natural language summaries for these RNAs where none previously existed. This is the first step to automating the summarization of literature in ncRNAs and providing helpful overviews to researchers.

## Supplementary Material

baaf006_Supp

## Data Availability

All code used to produce these summaries can be found at https://github.com/RNAcentral/litscan-summarization, and the dataset of contexts and summaries can be found at https://huggingface.co/datasets/RNAcentral/litsumm-v1.5. Summaries are also displayed on the RNA report pages in RNAcentral (https://rnacentral.org/) and can be explored by searching with the query ‘has_litsumm:“True”’ (https://rnacentral.org/search? q=has_litsumm:%22True%22).
