# Supplementary material for: LitSumm: large language models for literature summarization of noncoding RNAs
Source: Database (Oxford). 2025 Feb 5;2025:baaf006. doi: 10.1093/database/baaf006 (PMC11833236; doi:10.1093/database/baaf006)
Supplement: baaf006_Supp [file baaf006_supp.zip › suppl_data/Supplementary Materials.docx]

# Supplementary Material

### Appendix A

Below is the text contained within Figure 1 of the main text:

As an experienced academic who ALWAYS provides references for each sentence you write, produce a summary from the text below, focusing on {ent_id} and using the references for each sentence.

{context_str}

The reference for each sentence in the text is given at the end of the sentence, enclosed by [].

For example, the first sentence has the reference [{first_ref}].

References should only be provided at the end of sentences, and MUST follow the style in the context. Do not list references at the end of the summary.

You MUST provide at least one reference per sentence you produce.

Use only the information in the context given above. Start your summary with a brief description of {ent_id}, noting its type.

Use 200 words or less.

Summary:

Below is the text contained within Figure 2 of the main text:

Here is a bullet point list of statements about the entity {ent_id}:

{bullet_summary}

The summary was derived from the following context:
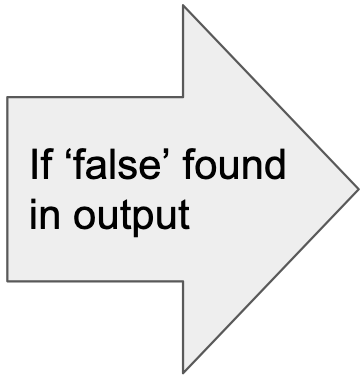


{original_context}

For each statement, determine whether it is true or false, based on whether there is supporting evidence in the context.

Make a determination for all statements, If a statement is false, explain why.

{checked_assertions}

In light of the above checks about its veracity, refine the summary below to ensure all statements are true.

Original summary:

{summary}

Do not change the reference style used, but you may add or remove references.

Revised summary:

Below is the text contained in Figure 4 of the main text:

LINC02499, a long intergenic non-coding RNA (lncRNA), has been observed to be significantly downregulated in hepatocellular carcinoma (HCC) tissues compared to adjacent non-tumor tissues, and its decreased expression is associated with poorer overall survival in HCC patients [PMC9775998], [PMC8831247], [PMC9720162]. This lncRNA is one of the top-ranking lncRNAs affecting the expression of cytochrome P450 family members CYP3A5 and CYP3A7, with a notable positive association with CYP3A expression [PMC9775998]. Despite its association with HCC prognosis and cytochrome P450 enzyme expression, the mechanisms by which LINC02499 regulates these processes remain to be elucidated [PMC9775998]. LINC02499 has been identified as a potential prognostic biomarker for HCC due to its significant correlation with clinical factors and overall survival, as well as its inclusion in prognostic models for HCC patients developed using LASSO Cox analysis [PMC9720162], [PMC8167205]. Furthermore, it has been shown to inhibit the proliferation, migration, and invasion of hepatocellular carcinoma cells in vitro [PMC9720162], suggesting a potential role as a tumor suppressor. However, further research is necessary to fully understand its function and clinical utility in HCC diagnosis and prognosis [PMC7812168].

Below is the text contained within Figure 5 of the main text:

- CTBP1-DT is a long non-coding RNA (lncRNA) whose expression is regulated by upstream open reading frames (uORFs) and internal ribosome entry site (IRES) regions within its 5′ untranslated region (5′UTR) [PMC9371908]

True. The context provided indicates that the uORFs and IRES regions within the 5′UTR of the CTBP1-DT gene are involved in the regulation of translation, which is consistent with the statement.

- These elements within the 5′UTR are crucial for the DNA damage-induced upregulation of CTBP1-DT translation [PMC9371908]

False. The context mentions "DNA damage-induced DDUP translation," not CTBP1-DT translation. There is no direct evidence in the provided context that these elements are crucial for the upregulation of CTBP1-DT translation specifically in response to DNA damage.

- Additionally, the expression levels of CTBP1-DT are influenced by the transcription factor ETV5, with changes in ETV5 levels either through knockdown or overexpression affecting CTBP1-DT expression [PMC8660778]

True. The context supports this statement by mentioning that the expression of lncRNA CTBP1-DT was measured following ETV5 knockdown or overexpression, implying that ETV5 levels have an effect on CTBP1-DT expression.

- This suggests a regulatory mechanism where ETV5 may play a role in modulating CTBP1-DT levels in response to DNA damage [PMC8660778].

False. The context provided does not mention DNA damage in relation to ETV5's role in modulating CTBP1-DT levels. The statement extrapolates beyond the provided evidence, which only indicates that ETV5 affects CTBP1-DT expression, not specifically in response to DNA damage.

Figure A1 below shows the four prompts used to rectify identified problems with the summary. Depending on the failure mode detected, the relevant prompt is dispatched to the LLM.

| **Adequacy**  The following summary:  {summary}  Does not have enough references. Add the correct references to each sentence, using only references from the following context:  {context_str}  References are provided in the context, enclosed in [].  For example, the first sentence has the reference [{first_ref}].  You must use the same format for references in the summary.  Revised Summary: | **Formatting**  The following summary:  {summary}  Uses the wrong format for references. Add the correct references to each sentence, using only references from the following context:  {context_str}  References are provided in the context, enclosed in [].  References in the summary that need replacing match the regex "\[\d+\]".  For example, the first sentence has the reference [{first_ref}].  You must use the same format for references in your summary.  Revised Summary: | **Realness**  The following summary:  {summary}  Contains references which do not appear in the context it was derived from.  Given the context below, revise the summary to include only references which appear in the context.  Context:  {context_str}  References are provided in the original context, enclosed in [].  For example, the first sentence has the reference [{first_ref}].  You should use the same format for references in your summary.  Revised Summary: | **Catchall**  Given the following summary:  {summary}  and its original context:  {context_str}  rewrite the summary to include at least one reference at the end of each sentence.  References are provided in the original context, enclosed in [].  For example, the first sentence has the reference [{first_ref}].  You should use the same format for references in your summary.  Revised Summary: |
| --- | --- | --- | --- |

*Figure A1: The four rescue prompts used to rectify problems with referencing in the generated summary. Depending on which of the checks fail, a different prompt is selected and used to modify the summary, with three of the four prompts having very specific instructions to the LLM, and the fourth being a generic catchall prompt when multiple failures are detected.*

We find it simpler to incorporate this kind of logic and multiple prompting than to design a single prompt that can ameliorate all failure modes.

## Appendix B

Human evaluation was carried out by four reviewers independently, using the rubric shown in Table B1, and a tool developed for the purpose (shown in figure B1). The tool allows raters to read the context from which the corresponding summary is generated, and give a rating of 1-5. There are also a series of checkboxes to identify common anticipated failure nodes, such as false positive IDs, or hallucinations.

*
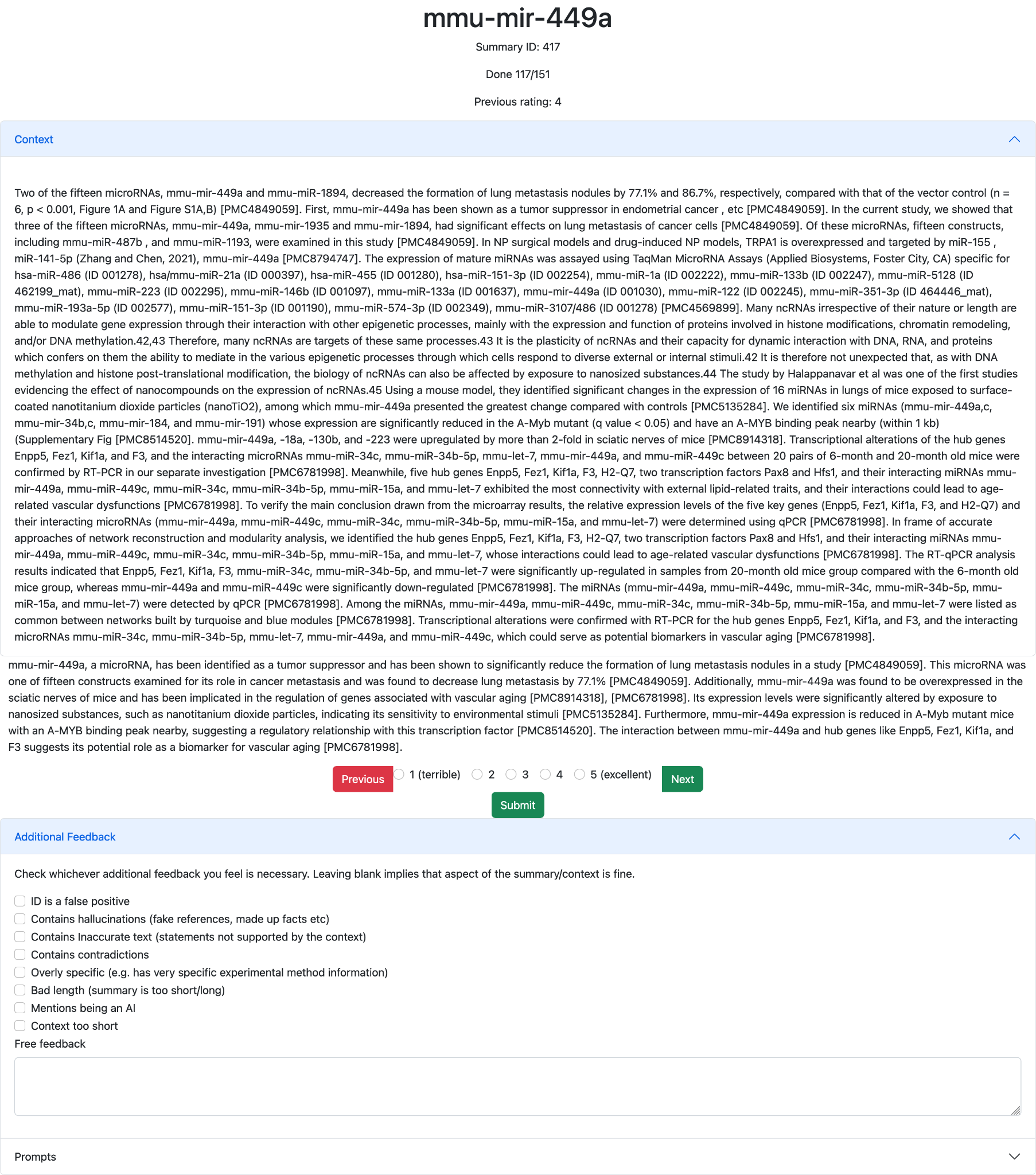
*

*Figure B1: A screenshot of the assessment tool used in the study. Raters are asked to read the shown summary and assess it given the information from the context. A score between 1 & 5 is given based on the rubric described in Table B1, and the yes/no questions are represented by tickboxes. Raters may optionally give free text feedback to explain their rating.*

Table B1 below shows the assessment rubric used by the human raters as they evaluated the summaries.

*Table B1: The assessment rubric used to evaluate summaries.*

| **Score** | **Criteria** |
| --- | --- |
| 5 | Excellent quality summary, as good or nearly as good as one written by a human curator. Fully referenced in the correct format, with all statements supported by the context. |
| 4 | A high quality summary, but with some small issues, for example style, flow, construction or missing information. All statements are factual and supported by the information in the context, references are real, adequate and presented correctly. Could be rescued by a few small changes |
| 3 | An acceptable summary. Style or flow may be of lower quality than a human might write. May miss considerable amounts of information. At most one incorrect or misleading statement. All references are real and given in the correct format. |
| 2 | A problematic summary with two incorrect/misleading statements, or one egregiously wrong statement. Large parts of the important information in the context are missing from the summary. References are given in the correct format, but may be inadequate. Style/flow may be poor. |
| 1 | Serious problems in the summary, for example:   - Incorrect reference format, total lack of references, restatement of references at the end of the summary - Hallucinated references, e.g. in author-year style - Multiple egregiously incorrect statements - Any mention of being an AI/LLM |

*Table B2: The tickboxes used for additional feedback on specific failure modes and the number of instances highlighted across all ratings by 4 raters. Due to our selection of RNAs starting from databases with well-defined IDs, there are no false positives identified. Similarly, our prompts are producing summaries that are a good length. However, there are a number of instances where the LLM has hallucinated or made misleading statements. On a few occasions, overly specific details, usually experimental techniques, are also mentioned in the summary. We also observe several instances of the summary mentioning, directly or indirectly, that it is being written by an AI which arise from the self consistency check.*

| **Factor** | **Instances (across 4 raters)** |
| --- | --- |
| ID is a false positive | 0 |
| Contains hallucinations (fake references, made up facts etc) | 8 |
| Contains Inaccurate text (statements not supported by the context) | 13 |
| Contains contradictions | 0 |
| Overly specific (e.g. has very specific experimental method information) | 3 |
| Bad length (summary is too short/long) | 0 |
| Mentions being an AI | 4 |
| Context too short | 1 |

## Appendix C

Below we give an example summary for some of the RNA types summaries in the paper. These come from our evaluation subset, and were the highest and lowest rated summary in each type

### miRNA

#### Best Summary

*MIR1249 is a microRNA that has been identified as one of the top up-regulated genes in certain cellular contexts [PMC9279949]. It has been implicated in the regulation of chemo-resistance, particularly through its role in the expansion of CD133+ cancer stem cells (CSCs) [PMC7590111]. Inhibition of MIR1249 has been shown to increase chemo-sensitivity by limiting the expansion of resistant cell populations, suggesting its potential as a therapeutic target [PMC7590111]. MIR1249 is also associated with Wnt signaling pathways, with pathway analysis indicating an enrichment in Wnt signaling upon MIR1249 upregulation [PMC7590111]. Clinically, high tumor expression of MIR1249 correlates with a worse prognosis independently of adjuvant chemotherapy [PMC7590111]. Furthermore, pathway analysis revealed that inhibition of MIR1249 affects pathways deregulated by chemotherapy, indicating its role in mediating chemo-resistance through these pathways [PMC7590111]. In vivo studies have confirmed that knockout (KO) cells for MIR1249 exhibit reduced tumorigenicity and increased sensitivity to chemotherapy, reinforcing its significance in cancer progression and treatment resistance [PMC7590111].*

#### Worst Summary

*hsa-mir-4270 is a microRNA whose expression is notably influenced by hsa_circ_0005556, as demonstrated by a significant upregulation in patient samples compared to controls [PMC7521983][PMC4867432]. Despite efforts, consistent expression patterns between qRT-PCR and microarrays for hsa-mir-4270 have not been established [PMC4658123]. This microRNA is implicated in various biological processes, including Acinar to Ductal Metaplasia (ADM) and has been identified as a potential exosomal miRNA (Exo-miRNA) [PMC9978013][PMC8104147]. Predictive models suggest that hsa-mir-4270 could act as a molecular sponge for other miRNAs, potentially regulating genes involved in SARS-CoV-2 infection mechanisms [PMC7521983][PMC9511898]. In the context of cancer, hsa-mir-4270's expression varies with cancer stage and has been identified as one of the most significantly upregulated miRNAs in breast cancer-associated studies [PMC4867432]. Additionally, it has been found to be upregulated in sepsis-induced Acute Kidney Injury (AKI) and differentially expressed between sepsis-induced AKI and non-AKI groups [PMC5351858]. Conversely, it is downregulated in leiomyoma compared to myometrium tissues [PMC9154092], highlighting its diverse roles across various diseases and conditions.*

Issues identified:

- Unclear where the expansion of the ADM acronym comes from
- Poor information synthesis
- Contains inferences unsupported by the context

### snoRNA

#### Best Summary

*SNORD111, a small nucleolar RNA (snoRNA), is implicated in the 2′-O-methylation of the G3929 nucleotide in 28S rRNA, a modification process that is essential for ribosomal RNA function [PMC9701848]. This snoRNA is transcribed from the intronic region of SF3B3 on chromosome 16q22.1 [PMC9701848]. Recent studies have highlighted SNORD111's upregulation in formalin-fixed paraffin-embedded (FFPE) tissues of clear cell renal cell carcinoma (ccRCC) patients [PMC7812721], as well as its significant upregulation in non-small cell lung cancer (NSCLC) patients compared to healthy donors, suggesting its potential as a non-invasive diagnostic biomarker for NSCLC and early-stage NSCLC [PMC9701848]. SNORD111's expression levels were found to be stable and consistently measurable in plasma, unaffected by Ribonuclease A treatment or extended storage, reinforcing its suitability for diagnostic applications [PMC9701848]. Despite these promising findings, the small sample size of studies limits the discovery of more cancer-related properties of SNORD111 and further research is needed to elucidate its role in tumorigenesis and potential clinical applications [PMC9701848].*

#### Worst Summary

*SNORD123 is a small nucleolar RNA (snoRNA) that is associated with the host gene SNHG18 and is involved in RNA modification processes [PMC9413531], [PMC4488298]. It exhibits a tissue-specific expression pattern, with strong expression in the lung and heart, and is implicated in various cellular functions, including autophagy and cell signaling [PMC1636440], [PMC4488298]. In colorectal cancer (CRC) cells, SNORD123's host gene-associated 5′-CpG islands are hypermethylated, which correlates with transcriptional silencing of the gene [PMC4581331], [PMC3495749]. This hypermethylation of SNORD123 has been observed across a wide spectrum of human cancer cell lines and primary tumors, suggesting that it may play a role in tumorigenesis through epigenetic modification [PMC3495749], [PMC5769367]. Moreover, patients with high expression levels of both SNHG18 and SNORD123 have been associated with shorter overall survival times in CRC, indicating its potential as a prognostic biomarker for cancer outcomes [PMC9413531]. Despite its observed downregulation in CRC cells, the exact biological functions of SNORD123 remain to be fully elucidated; however, it has been implicated as an important biomarker for cancer subtyping and prognosis in various tumor types including lung cancer and glioma [PMC6539089].*

Issues Identified:

- Contains inferences unsupported by the context

### lncRNA

#### Best Summary

*LINC01930, identified as a long intergenic non-protein coding RNA, has been implicated in schizophrenia, a finding supported by genome-wide association studies [PMC9917391]. This lncRNA is predominantly located in the nucleus and is not capable of encoding functional peptides, suggesting a regulatory role at the transcriptional level [PMC9917391]. Decreased levels of LINC01930 in the serum of schizophrenia patients have been observed and are negatively correlated with HERV-W ENV expression, indicating its potential as a serum-based biomarker for schizophrenia [PMC9917391]. LINC01930 appears to modulate the innate immune response by suppressing IFN-β production and inhibiting the cGAS signaling pathway activation mediated by HERV-W ENV in neuronal cells [PMC9917391]. Furthermore, LINC01930 has been shown to promote cell proliferation and reduce neuronal apoptosis induced by HERV-W ENV, suggesting its involvement in neuroprotection [PMC9917391]. Despite these findings, the precise role of LINC01930 in schizophrenia etiology remains to be fully elucidated [PMC9917391].*

#### Worst Summary

*LINC02499, a long intergenic non-coding RNA (lncRNA), has been observed to be significantly downregulated in hepatocellular carcinoma (HCC) tissues compared to adjacent non-tumor tissues, and its decreased expression is associated with poorer overall survival in HCC patients [PMC9775998], [PMC8831247], [PMC9720162]. This lncRNA is one of the top-ranking lncRNAs affecting the expression of cytochrome P450 family members CYP3A5 and CYP3A7, with a notable positive association with CYP3A expression [PMC9775998]. Despite its association with HCC prognosis and cytochrome P450 enzyme expression, the mechanisms by which LINC02499 regulates these processes remain to be elucidated [PMC9775998]. LINC02499 has been identified as a potential prognostic biomarker for HCC due to its significant correlation with clinical factors and overall survival, as well as its inclusion in prognostic models for HCC patients developed using LASSO Cox analysis [PMC9720162], [PMC8167205]. Furthermore, it has been shown to inhibit the proliferation, migration, and invasion of hepatocellular carcinoma cells in vitro [PMC9720162], suggesting a potential role as a tumor suppressor. However, further research is necessary to fully understand its function and clinical utility in HCC diagnosis and prognosis [PMC7812168].*

Issues identified:

- Incorrect references assigned
- Hallucinated details
- Statements unsupported by the context

## Appendix D

Originally, LitSumm was developed using GPT3.5 as the driving LLM. Comparing the results of automated checks between the two LLM versions shows the difference in the models’ capability, and the improvement of the GPT series over time. We also performed a simple A/B test to compare the summaries from the two models, which motivated the use of GPT4.

*Table D1: The error rates detected in the automated checking pipeline for GPT3.5 and 4*

| **Failure mode** | **GPT3.5 Pass Rate** | **GPT4 Pass Rate** |
| --- | --- | --- |
| References - first pass | 69% | 97.9% |
| References - after revision | 95% | 99.5% |
| Self-consistency - no problems found | 91% | 82.7% |
| Self consistency - no problems after revision | 97% | 91.5% |

Table D1 shows that for some aspects of self checking, GPT4 is clearly superior. The number of reference problems (e.g. inadequacy, formatting or location) if much reduced with GPT4 compared to GPT3.5, indicating the newer model’s superior ability to follow instructions.

Where the comparison is less clear is in the detection of self consistency errors, where the pass rates for GPT4 are markedly reduced. This is likely to be due to GPT4 being more able to identify self consistency errors, resulting in the reduced pass rate. However, GPT4 seems to be less adept at rewriting the summary after finding an error. This suggests that the output instructions from the model which it then follows are not well formed in some cases; investigating this aspect of the pipeline will form part of our future work.

Nonetheless, comparison between GPT4 and GPT3.5 has been found to favour GPT4 when three of the authors performed a blinded A/B test. As can be seen in Figure D1, GPT4 is preferred by all annotators in about 60% of cases, meaning it produced a summary that was easier to read, or more informationally complete. There was no strict rubric for this comparison, just a simple preference between two displayed summaries.


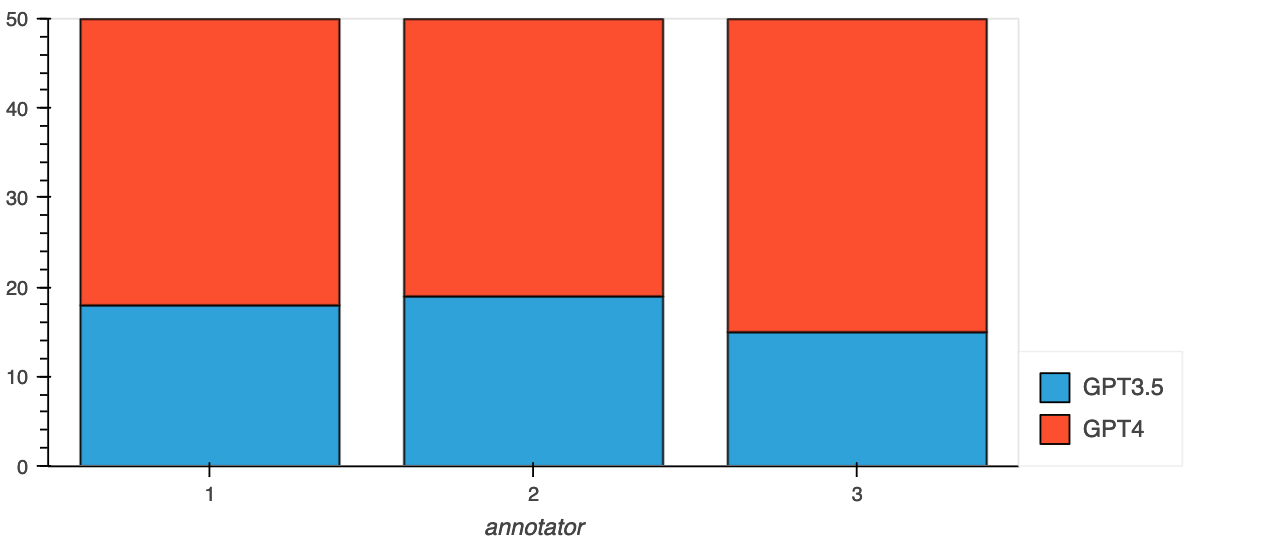


*Figure D1: A/B test comparing summaries from the GPT3.5 and GPT4.*
